# Emotional Reactivity and Regulation in Preschool-Age Children Who Do and Do Not Stutter: Evidence From Autonomic Nervous System Measures

**DOI:** 10.3389/fnhum.2020.600790

**Published:** 2020-12-16

**Authors:** Victoria Tumanova, Blair Wilder, Julia Gregoire, Michaela Baratta, Rachel Razza

**Affiliations:** ^1^Department of Communication Sciences and Disorders, Syracuse University, Syracuse, NY, United States; ^2^Department of Human Development and Family Science, Syracuse University, Syracuse, NY, United States

**Keywords:** cardiac vagal tone, RSA, skin conductance, heart rate preschool-age children, stuttering, emotional reactivity and regulation

## Abstract

**Purpose:** This experimental cross-sectional research study examined the emotional reactivity and emotion regulation in preschool-age children who do (CWS) and do not stutter (CWNS) by assessing their psychophysiological response during rest and while viewing pictures from the International Affective Picture System (Lang et al., 2008).

**Method:** Participants were 18 CWS (16 boys and two girls; mean age 4 years, 5 months) and 18 age- and gender-matched CWNS. Participants' psychophysiological responses were measured during two baselines and two picture viewing conditions. Skin conductance level (SCL) and heart rate were measured to assess emotional reactivity. Respiratory sinus arrhythmia (RSA) was measured to assess emotional regulation. Participants' shyness and executive function were assessed via parent report and considered for their effects on participants' psychophysiological responses.

**Results:** First, CWNS and CWS did not differ in their initial baseline SCL, heart rate, or RSA, but all participants had higher SCL and lower RSA in the second baseline, subsequent to the first challenge condition, compared to the first baseline. Second, during the challenge conditions, CWS did not differ from CWNS in their SCL, but showed a significantly higher heart rate than CWNS. Third, CWS exhibited a significantly lower RSA during the challenge conditions compared to CWNS. Lastly, the temperamental quality of shyness was associated with preschool-age children's SCL, such that participants who were rated higher in shyness had a higher SCL during the challenge conditions. Participants' executive function had a marginally significant effect on the RSA, such that the participants who had higher executive function composite scores exhibited lower RSA during the challenge conditions.

**Conclusions:** Our findings suggest that CWS and CWNS did not differ in their emotional reactivity and emotional regulation abilities at rest. During challenge conditions, however, CWS tended to be more emotionally reactive, as indicated by a higher heart rate, and also employed more emotional regulation, indexed by a greater decrease in RSA, compared to CWNS. Preschool-age children's behavior is largely dominated by reactivity, but there is the emergence of regulation, which can help children adjust to various contextual demands. For CWS who are more emotionally reactive, regulatory skills may be particularly critical to their prognosis and treatment.

## Introduction

Preschool age is the time of substantial growth in children's cognitive, motor, and social-emotional development. It is also the time when some children first show stuttering behaviors, which typically take the form of sound and syllable repetitions, prolongations, and tense pauses. Defined as involuntary disruptions to the rhythm of speech, stuttering affects about 5–11% of preschool-age children (Andrews and Harris, 1964; Yairi and Ambrose, 2005; Reilly et al., 2013). Research evidence and theoretical perspectives on stuttering suggest that it is a multifactorial neurodevelopmental disorder (Conture and Walden, 2012; Smith and Weber, 2017). Cognitive, linguistic, motor, and emotional factors have been theoretically and empirically linked to stuttering onset and development. This is not surprising, given the rapid development of these domains during the preschool years. The focus of this study is to identify emotional indicators involved in stuttering development. We examined situation-specific emotional reactivity and emotion regulation in 3–5-years old children who do and do not stutter during two picture viewing conditions. Children's emotional processes were assessed using electrodermal and cardiac measures of autonomic nervous system activity. Child temperament, which can influence how children experience emotional arousal, was assessed with a parent-report questionnaire. In the paragraphs below, we discuss the role of emotional processes for developmental outcomes, review research on the significance of these processes for stuttering development, and describe ways to objectively assess emotional processes in preschool-age children leading to the study's research questions and hypotheses.

Spoken communication is a complex process. It not only relies on precise coordination of respiration, phonation, and articulation, while simultaneously processing linguistic information, but also requires social engagement, which relies on the ability to regulate one's emotional arousal (Porges, 2003a; Garner and Waajid, 2012). In the field of psychology of emotions, an emotion is often defined as a configuration of peripheral physiological body changes, forms of expression, and subjective feeling states elicited by internal or external stimuli (Ekman, 1984; Scherer, 2005). Most emotions are short in duration compared to mood states, and could be viewed as biological signals because their experience and expression are associated with dynamically changing physiological activity. During the preschool-age years, with the rapid development of cognitive skills, emotions become increasingly accessible to regulation; that is, children can modify the intensity, duration, expression, and quality of their emotions.

An extensive body of research indicates that emotional reactivity and regulation in early childhood predict a broad array of outcomes, including the development of pro- or anti-social behaviors, academic competence and achievement, mental health, and overall healthy living. A comprehensive overview of the developmental outcomes influenced by children's emotional processes is available in several published meta-analytical studies (Allan et al., 2014; Compas et al., 2017; Smithers et al., 2018; Robson et al., 2020). Specific to spoken communication, a growing body of literature has revealed associations between temperamental traits and language development in very young children (Slomkowski et al., 1992; Dixon and Shore, 1997; Mundy and Gomes, 1998; Dixon and Smith, 2000; Morales et al., 2000; Kubicek et al., 2001; Salley and Dixon, 2007; Usai et al., 2009; Garello et al., 2012). As discussed by Salley and Dixon (2007), the emotional processes can impact speech and language both directly and indirectly. A heightened emotional state may place a burden on the child's behavioral control systems, leaving fewer resources to contribute to speech and language production, thereby directly affecting the child's language. Emotion can also have an indirect impact on language in that children who are more timid, shy, or anxious may limit their interactions and socializations with people around them, resulting in fewer opportunities for these children to practice and develop their language skills.

Emotional reactivity and emotion regulation are considered to be core components of temperament, which is defined as relatively stable, biologically-based, individual differences in reactivity and self-regulation (Derryberry and Rothbart, 1984). Within a neurobiological model of temperament (Derryberry and Rothbart, 1997), the body's defense and approach systems motivate adaptive behavior by inducing emotional states, while the executive attention system allows for the regulation or suppression of these reactive systems. Thus, temperament plays an important role in how an individual reacts to and interacts with their environment (Rothbart et al., 2000).

## Emotional Reactivity and Regulation in Children WHO Stutter

Emotional processes have also been considered in theoretical perspectives (e.g., Conture and Walden, 2012; Smith and Weber, 2017) and empirical research for their role in childhood stuttering development. Presently, there is no clear evidence that temperament plays a causal role in stuttering (Kefalianos et al., 2012; Alm, 2014) and lack of differences in temperament between CWS and CWNS have been reported (Reilly et al., 2013; Kefalianos et al., 2014, 2017). However, some converging research findings indicate that CWS may exhibit lower attentional control (e.g., Schwenk et al., 2007; Eggers et al., 2010, 2012, 2013), higher emotional reactivity (Anderson et al., 2003; Karrass et al., 2006; Choi et al., 2016), and greater negative affect than CWNS based on parent-report (Eggers et al., 2010; Ambrose et al., 2015) and direct behavior observation (Johnson et al., 2010; Ntourou et al., 2013). Two large scale United Kingdom and United States population studies found that preschool-age CWS were rated by their parents as more likely to have worries, be unhappy, and have difficulties with emotions (McAllister, 2016; Briley et al., 2019). Furthermore, per caregiver report, preschool-age CWS were significantly higher in behavioral inhibition, a correlate of shyness, than preschool-age CWNS (Ntourou et al., 2020; Tumanova et al., 2020a); however, studies using direct behavior observation of behavioral inhibition do not corroborate these findings (Choi et al., 2013; Tumanova et al., 2020a).

Although the research on the role of emotional reactivity and regulation in stuttering development remains inconclusive, is has been proposed that children who stutter who are more reactive and less able regulate their emotions may react to their disfluencies with stronger emotions, potentially leading to exacerbation of their stuttering, than children who stutter who are less reactive and more able to regulate their emotions (Conture and Walden, 2012; Guitar, 2019). Accordingly, some studies found associations between components of temperament and stuttering severity in young children who stutter (Schwenk et al., 2007; Boey, 2012; Choi et al., 2013; Kraft et al., 2014, 2019; Ntourou et al., 2020); others, however, did not observe these associations (Eggers et al., 2010; Tumanova et al., 2011; Alm, 2014).

Despite a growing number of studies that assessed emotional reactivity and regulation in children who stutter, the role of emotional processes in stuttering development remains unclear. One of the caveats of the existing research is that findings often come from parent-report rather than direct behavior observation or psychophysiological data. Given that the experience of emotion is associated with modulation in autonomic arousal, measurement of the autonomic nervous system activity in response to emotional stimuli offers a reliable means to objectively assess emotional reactivity and regulation in young children (Fowles et al., 2000; El-Sheikh, 2007; Kreibig, 2010). Through measurement of physiological autonomic nervous system processes we can capture emotional reactions that are covert or non-conscious. Given that physiological measures only provide a snapshot on how children perform in a novel laboratory environment, they can be used in conjunction with parent-report of children's behavioral tendencies to provide a more comprehensive assessment. This approach is especially beneficial when studying preschool-age children, whose young age precludes them from describing their personality and emotional states reliably. Thus, in this study, following established recommendations for multimethod assessment (Posner et al., 2014), we examined reactive and regulatory components of temperament via both psychophysiological assessment and parent report questionnaire. Emotional reactivity was assessed by measuring children's electrodermal activity and heart rate, and emotion regulation was assessed by measuring respiratory sinus arrhythmia (RSA) during rest and picture-viewing conditions.

Given their potential significance for childhood stuttering development, shyness and executive function were assessed for their effects on the autonomic nervous system response in preschool-age participants. The construct of shyness was chosen because it reflects a child's tendency to approach new situations and people. Two recent studies have demonstrated that preschool-age CWS are rated higher in shyness by their caregivers (Ntourou et al., 2020; Tumanova et al., 2020a). Temperamental quality of shyness has also been implicated in the way children who stutter respond in unfamiliar situations (Choi et al., 2013; Ntourou et al., 2020; Tumanova et al., 2020a). Lastly, shyness has been associated with higher levels of electrodermal activity in young children (Kagan et al., 1988; Scarpa et al., 1997).

The construct of executive function was chosen because it has been linked to childhood stuttering (Ntourou et al., 2018; Anderson and Ofoe, 2019). Executive function refers to a suite of higher-order cognitive processes, particularly attention, working memory, and inhibitory control, that are implicated in planning and goal-directed behavior (Miyake et al., 2000). Executive function skills develop rapidly across the preschool years (Garon et al., 2008; Hughes, 2011) and have been shown to interact with emotional reactivity (e.g., Blair and Raver, 2015) and with motor control (e.g., Becker et al., 2014) to influence a child's behavior. Research in the area of stuttering has linked executive function skills with both stuttering frequency and severity in young children (Kraft et al., 2014, 2019; Jones et al., 2017). Given that children who stutter (CWS) display deficits in executive function (Anderson and Wagovich, 2010; Eichorn et al., 2017), they may show heightened physiological responses to contextual stressors.

## Electrodermal Activity As a Measure of Emotional Reactivity

The autonomic nervous system consists of two branches, sympathetic and parasympathetic, that work reciprocally and continuously in response to stimuli in an effort to regulate the body (Gabella, 2012). Activity of the sympathetic branch of the autonomic nervous system is a major component in generating an emotional response. Specifically, the sympathetic nervous system is commonly known for generating the “fight or flight” response and reacting to environmental stimuli by increasing heart rate, contracting smooth muscles, expanding the lung bronchial tubes, signaling glands to release adrenaline and activating eccrine sweat glands in the skin (Dawson et al., 2007). Eccrine sweat glands are innervated solely by the sympathetic nervous system. When they are activated, they release sweat, which increases electrical conductance of the skin (Fowles, 1993). Palms and fingers have a high number of eccrine sweat glands. Thus, measuring the electrodermal activity on the surface of the palm or fingers allows for a reliable assessment of the sympathetic nervous system activity (Boucsein, 2012). Given the non-invasive nature of the electrodermal activity measurement, it has been widely used in developmental psychophysiology to examine children's responses to a variety of stimuli (Fowles et al., 2000; El-Sheikh, 2007; Kreibig, 2010; Nikoli et al., 2018).

Psychophysiological research with children who stutter is somewhat limited. However, recently a number of studies have examined CWS's autonomic nervous system response to such speaking conditions as picture naming, picture description, and non-word repetition (Jones et al., 2014, 2017; Zengin-Bolatkale et al., 2015, 2018; Choi et al., 2016; Tumanova and Backes, 2019; Walsh and Usler, 2019; Walsh et al., 2019). Results of these studies generally indicate that CWS do not have an elevated level of autonomic arousal during speaking. However, between-group differences in arousal, based on age (Zengin-Bolatkale et al., 2015) and task complexity (Tumanova and Backes, 2019), as well as within-CWS differences, based on stuttering chronicity (Zengin-Bolatkale et al., 2018), and speech fluency (Walsh et al., 2019) have been observed.

Fewer studies have attempted to manipulate physiological arousal by presenting emotionally evocative stimuli to examine whether differences in emotional reactivity and regulation would emerge between CWS and CWNS. One such study, conducted by Jones and colleagues (Jones et al., 2014), measured respiratory sinus arrhythmia (RSA; a measure of parasympathetic autonomic nervous system activity) and skin conductance level (SCL; which reflects continuous background activity of the sympathetic nervous system) in preschool-age children while they watched positively- and negatively-valenced video clips and completed picture description tasks immediately after video viewing. In regard to the skin conductance findings, they reported no differences between CWS and CWNS during rest (the baseline conditions). In video-viewing conditions, however, CWS, compared to CWNS, demonstrated higher SCL during the positively-valenced video, and lower SCL during the negatively-valenced video. Moreover, while speaking, CWS, compared to CWNS, demonstrated a higher SCL during picture description tasks subsequent only to viewing of a positively-valenced video clip, but not subsequent to viewing of negative or neutral video clips (neutral video clips were used to establish the baseline for autonomic nervous system measures). These findings indicate that there may be differences in physiological emotional processes between preschool-age CWS and CWNS. However, the Jones et al. (2014) is but one study that examined these processes in preschool CWS. Additional research is needed to outline potential differences in psychophysiological response to challenge conditions among preschool-age CWS and CWNS.

## Heart Rate As a Measure of Emotional Reactivity

The cardiovascular system is one prominent component of the autonomic nervous system. As emotions are associated with the activation of the autonomic nervous system, people experience changes in their cardiovascular system, such as an increase in their heart rate (Sonnemans and Frijda, 1994; Kreibig et al., 2007). In addition to electrodermal activity, heart rate can serve as a measure of emotional reactivity (Uy et al., 2013). Unlike electrodermal activity, which is controlled only by the sympathetic branch of the autonomic nervous system, heart rate is influenced by both sympathetic and parasympathetic branches of the autonomic nervous system. An increase in heart rate is seen when the sympathetic nervous system engages a “fight or flight” response. A decrease in heart rate is seen when the parasympathetic nervous system engages a “rest and digest” response that acts to regulate the emotional arousal. Children and adults increase their heart rate in response to a social-emotional challenge, or subjective feeling of anxiety (Bradley et al., 2001; Kudielka et al., 2004). In contrast, during periods of rest, heart rate tends to decrease (De Munck et al., 2008).

Although heart rate has been used extensively in psychophysiological research (see Lorber, 2004), presently there are no published studies that examine heart rate at rest or in challenge conditions in CWS. Heart rate has, however, been used to examine sympathetic arousal in adults who stutter (Peters and Hulstijn, 1984; Weber and Smith, 1990; Bauerly et al., 2019). Converging evidence from the three studies indicate no differences in heart rate between adults who do and do not stutter and suggest a uniform increase in heart rate from baseline to challenge conditions in both groups. Admittedly, findings observed in a mature system of adults who stutter cannot be extended to the rapidly developing system of preschool-age children.

## Respiratory Sinus Arrhythmia As a Measure of Emotion Regulation

Another measure of cardiovascular system that plays an important role in emotional processes, specifically in emotion regulation (Porges, 2009), is the respiratory sinus arrythmia (RSA). RSA reflects the variation in interbeat intervals of the heart at the frequency of breathing (Gentzler et al., 2009). It is controlled by the parasympathetic branch of the autonomic nervous system, which is responsible for keeping the body calm and conserving energy and resources. The parasympathetic nervous system influence on the heart operates through the vagus nerve and is often described as the vagal brake or vagal tone (Porges, 1995; Porges et al., 1996). When parasympathetic nervous system is active, the vagal activity (or vagal tone) is high, and the vagal brake is activated, which slows the heartbeat. In contrast, when vagal activity (or vagal tone) is low, the vagal brake is inactivated and heart rate can increase, allowing the body to respond to a stressor. The amplitude of RSA is one way to quantify the vagal tone (Porges et al., 1994).

According to the Polyvagal theory (Porges, 1995, 2003a,b, 2007), RSA is linked to social behavior, as our range of social behavior is limited by our physiological state. Higher RSA at rest would be adaptive as it reflects a greater capacity for self-regulation and social engagement. States of calmness are associated with more vagal control, and an overall higher RSA. In contrast, states of stress and vulnerability are associated with having less vagal control, an overall lower RSA. When a person must respond to a challenge, the vagal brake is withdrawn, thereby allowing heart rate to increase and the person to meet environmental demands (Porges et al., 1996).

A growing body of research suggests that individual differences in children's RSA are associated with their emotion regulation ability (Graziano and Derefinko, 2013; Compas et al., 2017). The RSA-related emotion regulation is typically assessed by measuring changes in RSA (i.e., vagal tone/vagal brake) between rest (or a baseline condition) and a challenge condition. Higher RSA at rest and a greater decrease in RSA during a challenge condition have been linked to more positive and less negative affect, and more effective emotion regulation strategies in children (e.g., Calkins, 1997; Gottman and Katz, 2002; Calkins and Keane, 2004; Calkins et al., 2007; Hessler and Fainsilber Katz, 2007; Santucci et al., 2008; Gentzler et al., 2009; Bal et al., 2010; Musser et al., 2011; Graziano and Derefinko, 2013).

Given that emotional regulation is implicated in childhood stuttering development, examination of changes in RSA from rest to emotionally arousing conditions in preschool-age CWS and CWNS can provide an insight into the role of emotional factors in stuttering development. Presently there are only two studies that investigated emotional reactivity using RSA in children who stutter (Jones et al., 2014, 2017). The Jones et al. (2014) study, described above, examined SCL and RSA in preschool-age CWS and CWNS during baseline, emotionally arousing video clips, and picture description tasks immediately following the video- viewing conditions. The RSA-specific findings indicated that during the baseline and picture description conditions CWS had significantly lower RSA than CWNS. However, there was no significant between-group difference for RSA during the video-viewing conditions.

In a closely related follow-up study, Jones et al. (2017) combined parent-report measures of the participants' executive function (per the Children's Behavior Questionnaire (CBQ, Rothbart et al., 2001) with measures of RSA during the same conditions as the ones in Jones et al. (2014) to examine whether preschool-age CWS and CWNS's RSA-based emotional regulation and executive function ability were associated with the frequency of their stuttered disfluencies during the picture description conditions. It was found that baseline RSA (at rest) was not associated with subsequent speech fluency for either CWS or CWNS. Interestingly, lower RSA (greater decrease from baseline) during the challenge conditions (video viewing and picture description) was related to higher levels of stuttering in both groups (CWS and CWNS). This pattern was observed even in CWNS, despite the fact that the frequency of stuttered disfluencies in the CWNS group was very low (1.7% stuttered disfluencies, which is below the stuttering diagnostic criterion of 3%). The authors interpreted the findings as suggesting that children who engage in higher emotional regulation during challenge conditions (as indexed by lower RSA) may have fewer resources to support social communication, resulting in lower fluency (higher frequency of stuttering). The Jones et al. (2017) study provides the initial evidence that physiological aspects of emotion regulation may be associated with stuttering. Their findings corroborate several behavioral studies that reported an association between emotional regulation processes and stuttering in children (Arnold et al., 2011; Ntourou et al., 2013; Jones et al., 2014).

### Purpose of the Study

The research over the last 20 years underscores the importance of emotional reactivity and regulation processes for early childhood developmental outcomes. Thus, our goal was to objectively assess emotional reactivity and regulation in preschool-age CWS and CWNS using psychophysiological measures. Extant literature on the emotional contributions to stuttering development remains inconclusive. However, initial evidence suggests that preschool CWS and CWNS may differ in their response to challenge conditions (Jones et al., 2014) and that RSA-based emotional regulation may be associated with stuttering frequency in preschool-age children (Jones et al., 2017). In the present study, we sought to further examine physiologically-based emotional reactivity and regulation during rest and emotionally arousing challenge conditions in preschool-age CWS and CWNS, and determine whether the physiological reactivity of preschool-age children is associated with parent-reported shyness and executive function. We posed the following research questions:

1. Do preschool-age CWS and CWNS differ in their resting physiological state?
2. Do preschool-age CWS have higher emotional reactivity during picture viewing conditions than CWNS?
3. Do preschool-age CWS exhibit lower emotional regulation during picture viewing conditions than CWNS?

Based on existing studies we hypothesized that (1) at rest, CWS would have an overall lower baseline RSA, and higher baseline HR as compared their typically developing peers, but there would be no differences in SCL between the groups; (2) during picture viewing, CWS would present an overall lower RSA, higher heart rate, and higher SCL compared to their typically developing peers; (3) all participants would exhibit higher SCL and heart rate and lower RSA during picture-viewing conditions than at rest.

Findings consistent with these predictions would indicate that the preschool-age CWS are particularly susceptible to the effects of their environment and may have difficulty regulating their body when in a state of physiological arousal during emotionally arousing conditions. Given that temperament has been shown to affect speech-language development in young children, a temperamental profile high in reactivity and low in regulation may contribute to the development of stuttering and to disruptions of speech fluency characteristic of stuttering (Conture and Walden, 2012).

## Methods

This study reports on the data collected from the same participants as those in the Tumanova et al. (2020a) study. Participants included 36 preschool-age children (age range: 38–69 months) and their caregivers. There were 18 CWS (16 boys and 2 girls; mean age 53.89 months or 4 years, 5 months) and 18 CWNS (16 boys and two girls; mean age 54.39 months or 4 years, 6 months). The majority of the children were white with the following racial breakdown by group (CWS: 15 Caucasians, two African Americans, and one multi-racial child; CWNS: 16 Caucasians, and two multi-racial children). All families were paid volunteers recruited through an advertisement in a monthly parent magazine circulated throughout Syracuse or an e-mail advertisement sent to Syracuse University employees. The study procedures were approved by the Syracuse University Institutional Review Board. Informed consent by parents and verbal assent by children were obtained.

### General Procedures

All experimental procedures and data collection occurred in the Syracuse University Stuttering Research Laboratory. Participants made two visits to the laboratory. During the first visit, participants were assessed for their speech, language and fluency skills, and during the second visit they completed the experimental tasks. The first visit to the lab started with a spontaneous conversation with the examiner elicited by age appropriate toys and free play. The conversation was recorded and analyzed for the frequency of stuttered disfluencies to determine group classification (please see the next section for details). Then, participants' speech articulation and language were screened with the “Sounds in Words” subtest of the Goldman-Fristoe Test of Articulation-2 (GFTA-2; Goldman and Fristoe, 2000) and Clinical Assessment of Language Fundamentals—Preschool 2 (CELF-P2; Wiig et al., 2004). All participants were within age-appropriate range of speech-language skills. Participants were also given a bilateral pure tone hearing screening at 20 dB loudness level to test frequencies of 1,000, 2,000, and 4,000 Hz (American Speech Language Hearing Association, 2015). Caregivers of all participants reported that their children had normal vision, English as the primary language, and no history of neurological diseases or diagnosed speech-language disorders apart from stuttering (for the participants in the CWS group).

#### Group Classification

Participants who (a) produced 3% or more of stuttered disfluencies (i.e., sound/syllable repetitions, sound prolongations, or monosyllabic whole-word repetitions) in a 300-word conversational speech sample during free play in visit one (Tumanova et al., 2014), (b) scored 10 or higher on the Stuttering Severity Instrument-4 (SSI-4; Riley, 2009), and (c) whose caregivers were concerned about their stuttering, were placed in the CWS group. Children who produced <3% stuttered disfluencies and whose caregivers showed no concern about their speech fluency were placed in the CWNS group. Stuttering frequency and severity characteristics for CWS are presented in Table 1.

**Table 1:** Stuttering severity, assessed by the stuttering severity instrument−4 (SSI-4; Riley, 2009), for children who stutter (CWS).

**Participant number**	**Group**	**Stuttering frequency (%)**	**SSI-4 score**	**Stuttering severity**
1	CWS	3	12	Mild
2	CWS	5	16	Mild-moderate
3	CWS	7	20	Moderate
4	CWS	4	12	Mild
5	CWS	4	14	Mild-moderate
6	CWS	5	12	Mild
7	CWS	8	18	Moderate
8	CWS	6	14	Mild-moderate
9	CWS	8	20	Moderate
10	CWS	7	18	Moderate
11	CWS	9	16	Mild-moderate
12	CWS	4	10	Very mild-mild
13	CWS	14	20	Moderate
14	CWS	5	14	Mild-moderate
15	CWS	4	10	Very mild-mild
16	CWS	6	16	Mild-moderate
17	CWS	3	10	Very mild-mild
18	CWS	4	16	Mild-moderate

#### Measure of Temperament

Participants' temperament was assessed with the Children's Behavior Questionnaire Short Form (CBQ, Rothbart et al., 2001; Putnam and Rothbart, 2006). The CBQ Short Form was completed by the caregiver (mothers in the majority of cases) who brought the child to the lab. The CBQ Short Form is a normed instrument with established validity and reliability that has been successfully used in other research on temperament and childhood stuttering (Eggers et al., 2010; Ambrose et al., 2015; Jones et al., 2017). The CBQ Short Form consists of 94 items scored on the seven-point Likert scale (1 = Extremely Untrue, 2 = Quite Untrue, 3 = Slightly Untrue, 4 = Neither True nor Untrue, 5 = Slightly True, 6 = Quite True, 7 = Extremely True) with a Not Applicable (N/A) option available. The caregiver rates their child's behavior on 15 different behavior dimensions. These 15 behavior dimensions combine to form three composite scores, the CBQ factors: (a) Surgency (activity level, approachability, high intensity pleasure, impulsivity, and shyness), (b) Negative Affectivity (anger/frustration, discomfort, fear, sadness, and soothability), and (c) Effortful Control (attentional focusing, inhibitory control, low intensity pleasure, perceptual sensitivity, smiling, and laughter).

Whereas, the entire CBQ Short Form was administered to assess the participants' temperament, we were specifically interested in the CBQ scale of Shyness, Inhibitory Control and Attention Focusing for their potential significance for childhood stuttering development. CBQ Shyness scale was chosen because if reflects a child's tendency to approach new situations and people. CBQ Inhibitory Control and Attention Focusing scales were chosen because they represent the cognitive skills consistent with executive function. Group scores on these three CBQ scales are presented in Table 4.

CBQ Inhibitory Control and Attention Focusing scales were significantly correlated in our sample (*r* = 0.366, *p* = 0.014). Given the interest in executive function skills in the area of stuttering, similar to Jones et al. (2017), we created an executive function composite score by averaging the scores on these two scales.

#### Emotion Elicitation Stimuli

To elicit emotional arousal, participants were shown 10 pictures with negative valence and 10 neutral pictures selected from the International Affective Picture System (IAPS; Lang et al., 2008). The IAPS was chosen because the subjective, psychophysiological, behavioral, and neurophysiological reactions elicited by the IAPS affective stimuli have been well-documented (for review see Lang and Bradley, 2007). Specifically, each IAPS photograph has an emotional valence rating made by men, women, and children and is standardized on the basis of ratings of pleasure/displeasure and level of arousal it elicits. Ten pictures with negative valence and 10 neutral pictures were selected to be age-appropriate and based on event-related potential studies of children's emotional processing using these specific pictures as stimuli (Hajcak and Dennis, 2009; Solomon et al., 2012). Five IAPS pictures were presented per one trial of a given condition. Two sets of IAPS pictures in each condition were balanced for their valence and arousal ratings and for content. Tables 2, 3 provide detailed information on the IAPS pictures used in this study.

**Table 2:** IAPS pictures presented to the participants in negative condition.

**Picture ID**	**Set**	**Valence**	**Arousal**	**Mean valence**	**Mean arousal**	**Difference significant**
Shark 1930	1	3.79 (3.91)	6.42 (7.71)	3.17	6.01	n.s.
Angry man 2120	1	3.34 (4.14)	5.18 (5.83)			
Snake 1120	1	3.79 (3.92)	6.93 (6.58)			
Soldier 9421	1	2.21 (4.19)	5.04 (5.56)			
Fire 8485	1	2.73	6.46			
Snake 1050	2	3.46	6.87	3.71	6.05	n.s.
Crying boy 2900	2	2.45	5.09			
Face painted man 2780	2	4.77 (6.00)	4.86 (5.69)			
Dog 1300	2	3.55 (4.11)	6.79 (7.11)			
Bear 1321	2	4.32	6.64			

*Ratings of valence and arousal taken from IAPS technical report (Lang et al., 2008). Rating are based on adult respondents (females and males). For selected pictures ratings by 7–9-year-old children were available, those are included in brackets*.

**Table 3:** IAPS pictures presented to the participants in neutral condition.

**Picture ID**	**Set**	**Valence**	**Arousal**	**Mean valence**	**Mean arousal**	**Difference Significant**
Towel 7002	1	4.97	3.16	4.92	2.43	n.s
Spoon 7004	1	5.04	2			
Bowl 7006	1	4.88	2.33			
Shoes 7031	1	4.52	2.03			
Book 7090	1	5.19 (5.97)	2.61 (3.11)			
Fire hydrant 7100	2	6.06 (6.06)	2.94 (2.94)	5.23	2.79	n.s.
Umbrella 7150	2	4.72 (5.89)	2.61 (2.75)			
Lamp 7175	2	4.87	1.72			
Watch 7190	2	5.55	3.84			
Chair 7235	2	4.96	2.83			

*Ratings of valence and arousal taken from IAPS technical report (Lang et al., 2008). Rating are based on adult respondents (females and males). For selected pictures ratings by 7-9-year-old children were available, those are included in brackets*.

#### Experimental Procedure

At the second visit to the lab, participants were seated in front of a computer screen. Hypoallergenic electrodes were attached to the skin of the distal phalanges of the index and middle finger of the left hand for acquisition of electrodermal activity, and to the skin at the suprasternal notch of the rib cage and at the 12th rib laterally to the left for acquisition of the electrocardiogram throughout the experimental tasks (Venables and Christie, 1980). Small movement sensors were also attached to the participants' lips and jaw and modified plastic goggles that participants wore during the experiment. The lip and jaw movement data were collected to address separate research questions about group differences in speech motor control (Tumanova et al., 2020a) and these data are not included in this report. E-prime software (2016, Psychology Software Tools, Inc.) was used to visually present the picture stimuli and to time-lock the picture presentations to the recorded physiological data.

**Figure 1:**
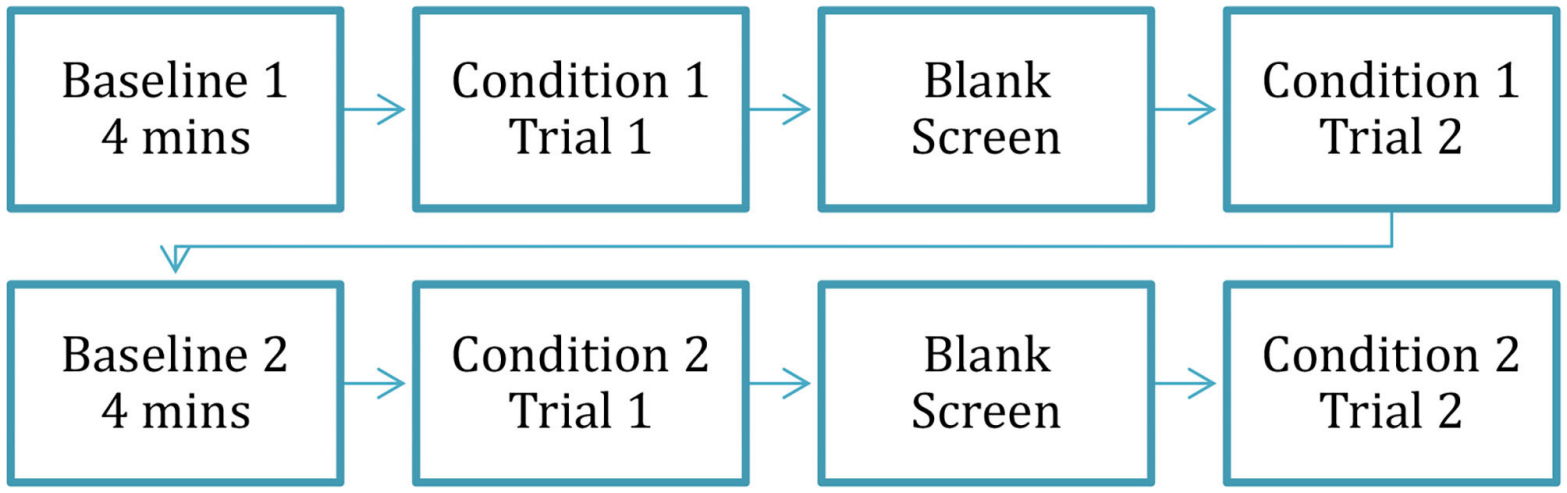
Time course of the entire experiment. Reprinted with permission from Tumanova et al. (2020b). Copyright 2020 American Speech-Language-Hearing Association.

Figure 1 provides an overview of the time course of the experimental conditions. First, to establish a pre-experimental baseline for each participant's resting skin conductance level and heart rate, participants viewed an animated screensaver of a three-dimensional fish tank for four min. The screensaver contained minimal action and had been previously successfully used to establish baseline levels of electrodermal activity and heart rate in preschool-age children (e.g., Jones et al., 2014; Tumanova and Backes, 2019). Next, the participants completed two experimental conditions: (1) viewing pictures with negative valence, and (2) viewing pictures with neutral valence. The order of the condition presentation (negative vs. neutral) was counterbalanced between the participants. Each experimental condition included two trials described in the following paragraph, comprising a total of four trials for the entire experiment (2 conditions × 2 trials per condition).

**Figure 2:**
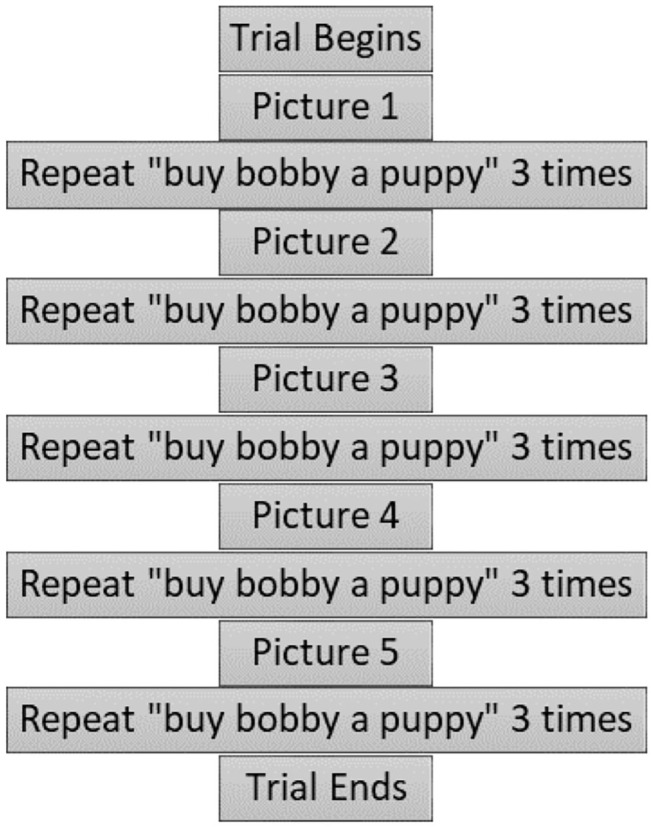
Time course of an individual trial. Reprinted with permission from Tumanova et al. (2020b). Copyright 2020 American Speech-Language-Hearing Association.

Each trial lasted ~4 min. Figure 2 shows the sequence of events for a trial. During each of the four trials, participants viewed five pictures (either negative or neutral in valence depending on the condition). The pictures were presented on a 27-inch (diagonal size) computer screen, positioned ~6 feet away at the participant's eye level, directly in front of them. A fixation cross preceded picture presentation to center participant's gaze at the center of the screen. Each of the pictures was displayed at 75% screen size (~20 inches in diagonal size) for 3 s. After viewing each picture, the participant was prompted to repeat a simple phrase (“Buy Bobby a puppy”) three times following presentations of a voice recording. These data are reported in a different publication (Tumanova et al., 2020a). For each trial, five different pictures were presented, and three repetitions of the target phrase were elicited, as shown in Figure 2.

After the completion of the first trial, participants were given a short break (~2 min) during which they were given a sticker. Then, the participants completed the second trial of the first condition. Finally, to re-establish the baseline for autonomic activity measurement before the second condition the participants viewed the animated screensaver again for 4 min. After the second baseline was completed, the second experimental condition was presented (as shown in Figure 1).

#### Measures of Electrodermal and Cardiac Activity

Electrodermal and cardiac activity was acquired using Biopac MP150 hardware system (Biopac Systems, Inc.) and analyzed using AcqKnowledge (ver. 4.3 for PC, Biopac), Cardioedit, and Cardiobatch software^1^ (Brain-Body Center, University of Illinois at Chicago).

Standardized procedures for electrodermal activity recordings were implemented (Boucsein et al., 2012). The electrodes were connected to a Biopac GSR100C skin conductance amplifier. The electrodermal activity (expressed in microSiemens, μS) was sampled at 1,250 Hz with the gain set at 10 μS/V and a low-pass filter at 1 Hz and subsequently downsampled for the analysis. The data were visually inspected during data collection to monitor for any instances of artifacts. To measure tonic arousal, mean skin conductance levels were calculated for the baselines and the picture viewing conditions using AcqKnowledge 4.3 software from a continuous electrodermal activity signal. Following common procedures (e.g., Boucsein et al., 2012), skin conductance level was calculated after phasic responses were removed from the signal.

Electrocardiogram was collected using a Biopac ECG 100C amplifier. The electrocardiogram (ECG) was sampled at 1,250 Hz and processed using the AcqKnowledge software. The raw ECG signal underwent a bandpass filter (0.5–35 Hz) to remove low frequency drift and high frequency noise. Then, AcqKnowledge software was used to detect the peak of the R-wave and obtain interbeat interval (IBI) time series (in milliseconds). The IBI time series were produced for each of the study conditions. The IBI waveforms were visually examined and edited for artifacts in Cardioedit software^2^ (Brain-Body Center, University of Illinois at Chicago, 2007) by two research assistants who completed and passed the required training associated with the software. No more than five percent of the total data for any one condition were corrected. CardioBatch software^1^ (Brain-Body Center, University of Illinois at Chicago, 2007) was used to calculate RSA and heart rate from corrected IBI time series. RSA was calculations were completed using the method developed by Porges (1985a). This method is mathematically equivalent to frequency domain methods for the calculation of the amplitude of RSA (Porges and Byrne, 1992). This is a multistep algorithm involving time sampling of the IBI waveform into 250-ms samples and applying a bandpass filter to extract variance based on the recurrence of respiration for young children (0.24–1.04 Hz). The amplitude of RSA is then calculated by taking the natural logarithm of the filtered time series [ln(ms)^2^]. For further details, please see the following publications (Porges, 1985b; Heilman et al., 2008; Lewis et al., 2012). Values of RSA were based on consecutive 30 s epochs within each condition (Heilman et al., 2012; Jones et al., 2014). The average of the 30 s epochs within each condition were used as the dependent variable in the statistical analyses.

### Reliability

Two research assistants who completed the required training associated with artifact correction using Cardioedit software^2^ (Brain-Body Center, University of Illinois at Chicago, 2007) visually examined and edited IBI time series from 12 participants (~30% of the data) independently. The reliability of measurement between the two research assistants was assessed by calculating intra-class correlation coefficients (ICC) using two-way mixed models and absolute agreement criterion (McGraw and Wong, 1996; Hallgren, 2012). The results of these comparisons indicated strong reliability for the first and second research assistant's RSA measurement (ICC = 0.997, *p* < 0.0001). The above ICC reliability values exceed the accepted criterion of 0.7 (Yoder and Symons, 2010).

### Dependent Measures

Skin conductance level (SCL) and mean heart rate served as objective measures of emotional reactivity. The RSA served as objective measures of emotional regulation.

### Statistical Analyses

Statistical analyses of the data were performed in IBM SPSS version 26 statistics software using linear mixed-effects models (Oleson et al., 2019). Each outcome variable (SCL, heart rate, and RSA) was tested in a separate model. Before conducting the main statistical analyses for each research question, distributions of each dependent variable were visually inspected with histograms and checked for normality based on descriptive statistics (mean, standard deviation, variance, skewness, and kurtosis). The alpha level was set to 0.05 for all models.

To examine physiological responses during rest (Research Question 1), we performed three general linear models with repeated measures on the Baseline (first, second). The statistical models included Group (CWS, CWNS) as a between-participant fixed factor, and Baseline (first, second) as a within-participant fixed factor. The models for Research Question 1 tested the main effects of Group and Baseline, and the interaction of Group × Baseline.

To examine physiological responses during the two picture viewing conditions (Research Questions 2 and 3), we performed linear mixed-effects models with repeated measures on Condition (neutral picture viewing, negative picture viewing) and Trial (first, second). Group (CWS, CWNS), and Order of Condition Presentation (explained below) served as between-participant fixed factors. The order of condition presentation (neutral vs. negative picture viewing) was counterbalanced between the participants (half of the participants in each group saw the neutral pictures first and half saw the negative pictures first). We previously reported (Tumanova et al., 2020a) that viewing negative pictures first had a priming effect on the level of arousal in the subsequent neutral condition, such that participants who started the experiment by viewing negative pictures had a higher level of arousal during the neutral picture viewing condition. Thus, we also included the order of the condition presentation as a fixed factor in the models.

### Co-variates

The law of initial values (Wilder, 1958) suggests that baseline physiological levels could influence physiological response in other experimental conditions. Thus, the first baseline SCL, heart rate, and RSA served as covariates for the models examining the SCL, heart rate, and RSA (respectively) during the picture viewing conditions. Age is known to be related to autonomic nervous system measures in children (El-Sheikh, 2005) and was entered as a covariate in all models. Lastly, CBQ Shyness score and executive function composite score served as covariates. Recall that CBQ Inhibitory Control and Attention Focusing scales were significantly correlated (*r* = 0.366, *p* = 0.014) in our sample, which is consistent with the results of others (Jones et al., 2017). Thus, to avoid collinearity in our statistical models, we created an executive function composite score, which served as a covariate in the models. Given the significant between-group differences in shyness and attention focusing, and reported effects of shyness and executive function on the autonomic nervous system measures, including these temperament-based variables as covariates served two purposes. It allowed us to examine the effects of these constructs on the physiological measures of autonomic nervous system activity and control for individual differences in these constructs among our participants.

The models for Research Questions 2 and 3 tested the main effect of Group, the interactions of Condition × Order of Condition Presentation, and Group × Condition × Order of Condition Presentation, the main effect of Trial, and the main effects of the four covariates (Baseline level of each autonomic nervous system measure, age, CBQ Shyness, executive function composite score). Tests for the main effects and interactions from the linear mixed-effects models were completed using Type III *F* tests with a Satterthwaite approximation for the degrees of freedom.

## Results

### Shyness and Executive Function

Group differences in CBQ Shyness and CBQ factor scores that formed the executive function composite score were examined in a multivariate general linear model. The model tested for the effect of Group (CWS, CWNS) on the three dependent variables, CBQ Shyness, CBQ Attention Focusing and CBQ Inhibitory Control. The results indicated a significant group difference in CBQ Shyness [*F*_(1, 34)_ = 4.262, *p* = 0.047, $\eta_{\text{p}}^{2}$ = 0.111], and CBQ Attention Focusing [*F*_(1, 34)_ = 5.789, *p* = 0.022, $\eta_{\text{p}}^{2}$ = 0.145]. Caregivers of CWS rated their children significantly higher in shyness and significantly lower in attention focusing than caregivers of CWNS. The group difference in CBQ Inhibitory Control was not significant [*F*_(1, 34)_ = 0.023, *p* = 0.880, $\eta_{\text{p}}^{2}$ = 0.001]. The descriptive statistics for the CBQ scores are presented in Table 4.

**Table 4:** Group differences in parent report of shyness and executive function-related skills per CBQ Short Form.

**Characteristic**	**Group**	**Mean**	**Std**	***N***	**Difference significant**
Shyness scale	CWNS CWS	3.33 4.22	1.2 1.38	18 18	*p* = 0.047
Executive function composite score	CWNS CWS	5.06 4.71	0.76 1.00	18 18	*n.s*.

*Std, standard deviation; executive function composite score is the average of attention focusing and inhibitory control scale scores*.

### Resting Physiological State

Descriptive statistics and Cohen's d effect size measures for the group differences in the outcome variables are presented in Table 5.

**Table 5:** Descriptive statistics for the dependent variables by group (18 CWS; 18 CWNS).

**Dependent variable**		**Group**	**Mean**	**Std**	**Effect Size Cohen's *d* (interpretation)**
Baseline 1 SCL		CWNS	9.31	5.57	*d = −0.54 (medium)*
		CWS	12.54	6.34	
Baseline 2 SCL		CWNS	14.21	4.60	*d* = –0.42 *(medium)*
		CWS	16.66	6.81	
Baseline 1 heart rate		CWNS	93.46	9.39	*d* = –0.23 *(small)*
		CWS	95.66	9.38	
Baseline 2 heart rate		CWNS	94.05	8.88	*d* = –0.26 *(medium)*
		CWS	96.48	9.63	
Baseline 1 RSA		CWNS	6.81	0.74	*d* = 0.27 *(medium)*
		CWS	6.57	1.03	
Baseline 2 RSA		CWNS	6.63	0.75	*d* = 0.25 *(medium)*
		CWS	6.41	1.00	
Neutral condition SCL	Trial 1	CWNS	14.91	5.80	*d* = –0.47 *(medium)*
		CWS	17.75	6.21	
	Trial 2	CWNS	15.63	5.30	*d* = –0.49 *(medium)*
		CWS	18.42	6.17	
Negative condition SCL	Trial 1	CWNS	14.72	6.29	*d* = –0.48 *(medium)*
		CWS	17.80	6.55	
	Trial 2	CWNS	15.24	5.54	*d* = –0.55 *(large)*
		CWS	18.50	6.27	
Neutral condition heart rate	Trial 1	CWNS	93.06	8.65	*d* = –0.62 *(large)*
		CWS	98.46	8.70	
	Trial 2	CWNS	95.18	7.86	*d* = –0.64 *(large)*
		CWS	100.25	8.07	
Negative condition heart rate	Trial 1	CWNS	93.64	8.54	*d* = –0.41 *(medium)*
		CWS	97.34	9.30	
	Trial 2	CWNS	94.81	8.75	*d* = –0.58 *(large)*
		CWS	99.96	9.10	
Neutral condition RSA	Trial 1	CWNS	6.65	0.76	*d* = 0.51 *(medium)*
		CWS	6.22	0.91	
	Trial 2	CWNS	6.59	0.83	*d* = 0.74 *(large)*
		CWS	5.93	0.94	
Negative condition RSA	Trial 1	CWNS	6.61	0.84	*d* = 0.19 *(small)*
		CWS	6.43	1.04	
	Trial 2	CWNS	6.45	0.72	*d* = 0.28 *(medium)*
		CWS	6.22	0.92	

*Std, standard deviation*.

#### SCL

Model results for SCL show that there was neither a main effect of Group [*F*_(1, 34)_ = 2.331, *p* = 0.136, $\eta_{\text{p}}^{2}$ = 0.064], nor a Group x Baseline interaction [*F*_(1, 34)_ = 0.391, *p* = 0.536, $\eta_{\text{p}}^{2}$ = 0.011] but there was a significant main effect of Baseline [*F*_(1, 34)_ = 53.177, *p* < 0.0001, $\eta_{\text{p}}^{2}$ = 0.610]. This indicates that CWS and CWNS did not differ in their SCL during either the first or second baselines, but all participants had lower SCL in the first baseline compared to the second baseline. Estimated marginal means for baseline SCL are 11.76 for CWNS, 95% CI [9.08, 14.44] and 14.60 for CWS, 95% CI [11.92, 17.28].

#### Heart Rate

Model results for baseline heart rate showed neither a main effect of Group [*F*_(1, 34)_ = 0.583, *p* = 0.450, $\eta_{\text{p}}^{2}$ = 0.017], Baseline [*F*_(1, 34)_ = 1.049, *p* = 0.313, $\eta_{\text{p}}^{2}$ = 0.030], nor a Group x Baseline interaction [*F*_(1, 34)_ = 0.025, *p* = 0.876, $\eta_{\text{p}}^{2}$ = 0.001]. These results indicate that CWS and CWNS did not differ in their mean heart rate during either first or second baselines and that there were no differences in heart rate between the first and the second baselines for both groups. Estimated marginal means for baseline heart rate are 93.76 for CWNS, 95% CI [89.40, 98.11] and 96.07 for CWS, 95% CI [91.72, 100.43].

#### RSA

Model results for RSA show that there was neither a main effect of Group [*F*_(1, 34)_ = 0.665, *p* = 0.420, $\eta_{\text{p}}^{2}$ = 0.019], nor a Group x Baseline interaction [*F*_(1, 34)_ = 0.002, *p* = 0.966, $\eta_{\text{p}}^{2}$ <0.0001] but there was a significant main effect of Baseline [*F*_(1, 34)_ = 7.161, *p* = 0.011, $\eta_{\text{p}}^{2}$ = 0.174]. Similar to the baseline SCL findings, these results indicate that CWS and CWNS did not differ in their RSA during either first or second baselines, but all participants had lower RSA in the second baseline compared to the first baseline. Estimated marginal means for baseline RSA are 6.73 for CWNS, 95% CI [6.31, 7.14] and 6.49 for CWS, 95% CI [6.07, 6.91].

### Physiological Reactivity during the Challenge Conditions (Picture-Viewing)

#### SCL

The linear mixed-effects model results show that there was a significant Condition x Order of Condition Presentation interaction [*F*_(3, 100)_ = 6.05, *p* = 0.001], in the absence of a significant Group x Condition x Order of Condition Presentation interaction [*F (*_3, 107)_ = 1.502, *p* = 0.218]. These results suggest that viewing negative pictures first had a priming effect on all participants' responses to neutral pictures. Those who viewed negative pictures first had a higher SCL during subsequent neutral picture-viewing condition (β = 1.92) than those who started with neutral pictures (β = −1.31) and then viewed the negative pictures (β = 0.74). Further, there was no effect of Group [*F*_(1, 130)_ = 0.006, *p* = 0.938, β = −1.827 for CWNS] or Trial [*F*_(1, 130)_ = 1.37, *p* = 0.244, β = −0.657 for Trial 1], but there was a significant effect of Age [*F*_(1, 130)_ = 7.697, *p* = 0.006, β = −0.10]. Younger participants had higher SCL during the picture viewing conditions. CBQ Shyness score also had a significant effect [*F*_(1, 130)_ = 4.518, *p* = 0.035, β = 0.49], such that participants who were rated higher in shyness showed a higher SCL during the picture viewing conditions. The executive function composite score was not significant in the model [*F*_(1, 130)_ = 1.741, *p* = 0.189, β = 0.45]. As expected, there was a significant main effect of baseline SCL [*F*_(1, 131)_ = 256.137, *p* < 0.0001, β = 0.809] on the SCL during the picture viewing conditions, those participants who had a higher baseline 1 SCL, had a higher SCL during the picture viewing conditions. Estimated marginal means for SCL during the challenge conditions are 16.60 for CWNS, 95% CI [15.76, 17.43] and 16.65 for CWS, 95% CI [15.81, 17.48].

#### Heart Rate

Model results for the heart rate during the picture-viewing conditions showed that there was a significant main effect of Group [*F*_(1, 130)_ = 12.087, *p* = 0.001, β = −3.652] with CWS exhibiting higher heart rate compared to CWNS, and Trial [*F*_(1, 130)_ = 7.366, *p* = 0.008, β = −1.92], with participants exhibiting higher heart rate during the second trial. As expected, there was a significant main effect of baseline heart rate [*F*_(1, 130)_ = 392.498, *p* < 0.0001, β = 0.85] on the heart rate during the picture-viewing conditions. The effects of Age (F (1,130) = 0.308, p = 0.580, beta = 0.024), CBQ Shyness score (*F* (1,130) = 2.313, *p* = 0.131, β = 0.457) and the Executive function composite score (*F* (1,130) = 1.190, *p* = 0.277, β = 0.468) were not significant in the model. The interactions of Group × Condition × Order of Condition Presentation [*F*_(3, 101)_ = 1.646, *p* = 0.183] and Condition × Order of Condition Presentation were not significant [*F*_(3, 90)_ = 1.22, *p* = 0.307], indicating that CWS and CWNS responded similarly to the two picture-viewing conditions and that the negative and the neutral picture viewing conditions elicited similar mean heart rates. Estimated marginal means for the heart rate during the challenge conditions are 95.23 for CWNS, 95% CI [94.19, 96.27] and 97.94 for CWS, 95% CI [96.90, 98.98].

### Physiological Regulation during the Challenge Conditions (Picture-Viewing)

#### RSA

Model results show that there was a significant main effect of Group [*F*_(1, 125)_ = 7.57, *p* = 0.007, β = 0.181], with CWS exhibiting lower RSA compared to CWNS, and Trial [*F*_(1, 124)_ = 4.641, *p* = 0.033, β = 0.18], with all participants exhibiting lower RSA in the second trial of picture viewing conditions. Executive function also had a marginally significant effect of the RSA [*F*_(1, 123)_ = 3.626, *p* = 0.059, β = −0.10], with participants who had higher executive function composite scores exhibiting lower RSA during picture viewing. CBQ Shyness was not significant in the model [*F*_(1, 123)_ = 2.007, *p* = 0.159, β = 0.05]. As expected, there was a significant main effect of the baseline RSA [*F*_(1, 123)_ = 276.857, *p* < 0.0001, β = 0.832]. The interaction of Condition × Order of Condition Presentation [*F*_(3, 88)_ = 2.192, *p* = 0.095] and the interaction of Group × Condition × Order of Condition Presentation were not significant in the model [*F*_(1, 92)_ = 2.125, *p* = 0.102], indicating that CWS and CWNS responded similarly to the two picture-viewing conditions and that the negative and the neutral picture viewing conditions elicited similar levels of RSA. Lastly, the effect of Age was not significant [*F*_(1, 122) =_0.178, *p* = 0.647]. Estimated marginal means for RSA during the challenge conditions are 6.51 for CWNS, 95% CI [6.39, 6.63] and 6.26 for CWS, 95% CI [6.14, 6.39].

## Discussion

This study expanded the literature on the role of emotional reactivity and regulation in preschool-age stuttering as reflected in four main findings. First, CWNS and CWS did not differ in the rest SCL, heart rate, or RSA (during baseline). Additionally, compared to the first baseline, both groups increased their sympathetic nervous system activity (higher SCL) and decreased their parasympathetic nervous system activity (lower RSA) during the second baseline, after the first challenge condition had been presented. Second, during the challenge conditions, CWS did not differ from CWNS in their SCL, but showed a significantly higher heart rate than CWNS. Third, CWS exhibited a significantly lower RSA during the challenge conditions compared to CWNS. Fourth, the temperamental quality of shyness was associated with preschool-age children's SCL, such that participants who were rated higher in shyness had a higher SCL during the challenge conditions. Participants' executive function had a marginally significant effect on the RSA, such that the participants who had higher executive function composite scores exhibited lower RSA during the challenge conditions. These findings are discussed below.

### Baseline Reactivity and Regulation in CWS

Our results indicated that preschool-age CWS and CWNS do not differ in their baseline SCL, heart rate or RSA, indicating that the two groups have a similar level of autonomic nervous system activity at rest. To our knowledge, this is the first study that examined the heart rate during rest and emotionally arousing conditions in preschool-age CWS. However, our findings with the other autonomic nervous system measures (SCL and RSA) corroborate the existing psychophysiological studies of preschool-age CWS. Specifically, a recent study from our laboratory (Tumanova and Backes, 2019) and the studies of others (Jones et al., 2014; Zengin-Bolatkale et al., 2015) reported no significant differences in baseline SCL between preschool-age CWS and CWNS.

According to the Polyvagal Theory (Porges, 2009), children who demonstrate lower RSA at rest experience more vulnerability to emotional reactivity and less emotional regulation. Following this theory, our baseline RSA results indicate that preschool-age CWS are equally prone to emotional reactivity and have the same emotional regulation ability as their typically fluent peers. Although these results did not support our hypothesis, they are consistent with the findings of Jones et al. (2017), who also found that preschool-age CWS and CWNS did not differ in their RSA during baseline.

Results from our study also indicated that all preschool-age participants (regardless of whether they stuttered or not) had a decrease in RSA from baseline 1 to baseline 2. This finding is consistent with that of Jones et al. (2014) who also observed a decrease in RSA in preschool-age CWS and CWNS from the first baseline (before an emotionally arousing condition) to the second baseline (after the emotionally arousing condition). Taken together and interpreted within the Polyvagal Theory, the findings discussed above suggest that CWS and CWNS experience equal proclivity for emotional reactivity and have equal ability for emotional regulation based on psychophysiological data.

### Emotional Reactivity During the Challenge Conditions

Our second research question was whether preschool-age CWS experience a higher emotional reactivity during emotionally-arousing picture viewing conditions than CWNS. Our results showed that CWS did not differ from CWNS in their SCL during the challenge conditions, but they had a significantly higher heart rate than CWNS, suggesting that they experienced a higher level of arousal. Only one other published study to date (Jones et al., 2014) attempted to manipulate emotional arousal of preschool-age CWS to examine autonomic nervous system measures of emotional reactivity and regulation. Jones et al. (2014) findings were somewhat inconsistent as they reported that CWS, compared to CWNS, demonstrated a higher SCL during the positively-valenced video, but a lower SCL during the negatively-valenced video. While speaking, CWS, compared to CWNS, only demonstrated a higher SCL during picture description tasks subsequent to viewing of a positively-valenced video clip, but not subsequent to viewing of negative or neutral video clips.

Although in the present study we did not find a significant between-group difference in SCL, our data showed a trend of higher SCL in CWS compared to CWNS during the two challenge conditions, which is supported by the medium to large effect sizes (Table 5). The observed SCL trend, taken together with the findings of a significantly higher heart rate in CWS during the challenge conditions, suggests that preschool-age CWS may have experienced a higher level of arousal associated with higher emotional reactivity compared with their peers who did not stutter. Admittedly, Jones et al. (2014) and this study do not provide conclusive evidence of higher emotional reactivity in preschool-age CWS. However, the combined findings outline potential differences in psychophysiological response to challenge conditions among preschool-age CWS and CWNS, which warrants further investigation.

Our data also suggest that preschool-age children's temperamental quality of shyness is associated with the sympathetic nervous system response to the challenge conditions. These findings are consistent with other published studies that examined this temperamental construct and its effect of the electrodermal activity in young children (Kagan et al., 1988; Scarpa et al., 1997). Temperament affects how children respond to and interact with their environment. Given the published evidence that the behavior inhibition trait (a correlate of shyness) has important implications for speech and language characteristics in preschool-age CWS (Choi et al., 2013; Ntourou et al., 2020; Tumanova et al., 2020a), studies that examine autonomic nervous system activity in CWS may consider including behavioral measures of shyness to account for the known variability in CWS's performance on many psychophysiological measures.

### Emotional Regulation During the Challenge Conditions

During emotionally arousing picture viewing conditions, we found that CWS exhibited significantly lower RSA than CWNS. Our data suggest that CWS in the present study experienced a higher level of arousal (greater emotional reactivity) during the challenge conditions than CWNS. Given these findings, it is logical to suggest that the lower RSA for CWS may represent an adaptive physiological response to the challenge conditions. Higher RSA at rest and a greater decrease in RSA during a challenge condition have been linked to positive affect and more effective emotion regulation strategies in children (e.g., Calkins, 1997; Gottman and Katz, 2002; Calkins and Keane, 2004; Hessler and Fainsilber Katz, 2007; Santucci et al., 2008; Gentzler et al., 2009; Bal et al., 2010; Musser et al., 2011; Graziano and Derefinko, 2013). According to the Polyvagal Theory (Porges, 2007), a well-regulated autonomic nervous system exhibits reciprocal activity, when sympathetic branch of the autonomic nervous system is activated, the parasympathetic branch is deactivated. Following this interpretation, our results indicate that CWS were experiencing an adaptive response to the challenge conditions, which elicited higher emotional reactivity in CWS than in CWNS peers. Our findings, partially corroborate those of Jones et al. (2014), who found that preschool-age CWS had higher level of arousal (higher SCL) coupled with lower RSA (suggesting higher emotional regulation), but only during the speaking conditions following video-viewing. No between-group difference in RSA was identified during the video-watching conditions. Importantly, in their closely related subsequent study Jones et al. (2017) found that CWS exhibited lower RSA during both the video viewing task and the subsequent speaking task. Further, their subsequent examination of the association between RSA level and frequency of stuttering (Jones et al., 2017) suggests that greater decrease of RSA during the emotionally arousing video viewing conditions (consistent with emotional regulation process) was related to more stuttered disfluencies during CWS's narratives that followed each condition. In light of these findings, for CWS, lower RSA likely manifests itself as greater vulnerability to emotional reactivity. As young children who stutter have been shown to have less stable speech motor control (Smith et al., 2012; MacPherson and Smith, 2013), the process of regulation of this higher reactivity (as indexed by the lower RSA) may take away resources from speech-language production leading to difficulties with maintaining fluency.

Lastly, our findings suggest that children's executive function skills may affect their ability to regulate their arousal. The participants who had higher executive function composite scores exhibited lower RSA during the challenge conditions, although this effect was only marginally significant. Research suggests that preschool-age CWS may have weaker executive function skills than CWNS (Ntourou et al., 2018; Anderson and Ofoe, 2019). The executive function skills have also been associated with stuttering frequency and severity in young children (Kraft et al., 2014, 2019; Jones et al., 2017). Given these findings, further research is needed to examine the link between low RSA during a challenge condition and executive function skills of young CWS. If proven significant, this association could provide insights into the physiological bases of known situational variability of stuttering and could have clinical implications.

Overall, these results indicate that though CWS and CWNS do not differ in their ability to regulate their emotions at rest, in situations where preschool-age children are emotionally stimulated, CWS may be more vulnerable to emotional reactivity than CWNS. This argument is supported by studies that have used caregiver questionnaires to measure emotional reactivity and regulation and found children who stutter to be more emotionally reactive than their CWNS peers. Both physiological data and parent-report data point to the fact that children who stutter may be more reactive to their environment than their typically developing counterparts. When in an arousing situation, these children may have fewer resources to devote to language and speech production, which could lead to more frequent speech disruptions in the form of stuttering disfluencies.

### Limitations

One of our study limitations is a relatively small sample size. A larger sample of preschool-age CWS and CWNS would have increased the power of our study and our ability to detect significant differences in the data. Further, our findings indicated that all participants regardless of group responded similarly to the negative and neutral pictures. The negative and the neutral picture viewing conditions elicited a similar level of SCL, RSA and heart rate indicating that sympathetic and parasympathetic activity did not change depending on which set of pictures participants were viewing. These results were unexpected given that the negative and neutral pictures were selected based on the valence and arousal ratings specified for each picture in IAPS technical report (Lang et al., 2008). We hypothesized that negative pictures would elicit a stronger physiological response (higher SCL and lower RSA) compared to the neutral pictures, which was not observed in our study. This study also employed parent-report measures of shyness and executive function, which may be less objective than direct assessments of these constructs.

## Conclusion

This study provides a physiological-based, objective look into the emotional processes that are thought to contribute to childhood stuttering development. Our findings suggest that CWS and CWNS did not differ in their emotional reactivity and emotional regulation abilities at rest. During challenge conditions, however, CWS tended to be more emotionally reactive, as indicated via higher heart rate, and also employed more emotional regulation, indexed by a greater decrease in RSA, compared to CWNS. The preschool years represent a unique period of social and cognitive development. Although children's behavior is largely dominated by reactivity, there is the emergence of regulation, which can help children adjust to various contextual demands.

We did not observe lower baseline RSA in CWS, which is promising since the Polyvagal Theory (Porges, 2009) suggests that low baseline RSA can negatively impact the ability to engage in social behaviors. The Polyvagal theory further suggests that when children are in an emotionally reactive state, systems important for social interaction, such as attention and speech production, may not be as supported as necessary. If some children who stutter are prone to high reactivity, regulatory skills may be particularly critical to their prognosis and treatment. Thus, emotional reactivity and regulation has clinical significance for preschool-age stuttering and should be considered during assessment and treatment of stuttering in children.

## Data Availability Statement

The original contributions generated for the study are included in the article, further inquiries can be directed to the corresponding author/s.

## Ethics Statement

The study procedures were reviewed approved by the Syracuse University Institutional Review Board. Written informed consent to participate in this study was provided by the participants' legal guardian/next of kin. Participants in this study, who were preschool-age children, provided verbal assent.

## Author Contributions

VT designed and supervised the experiment, participated in data collection and analysis, and wrote the manuscript. BW conducted data analysis and contributed to writing the manuscript. JG conducted data analysis and edited the manuscript. MB participated in data collection and analysis, and edited the manuscript. RR contributed to data interpretation, the writing and reviewing of the manuscript. All authors contributed to the article and approved the submitted version.

## Conflict of Interest

The authors declare that the research was conducted in the absence of any commercial or financial relationships that could be construed as a potential conflict of interest.

## Footnotes

^1^Cardiobatch software (Brain-Body Center, University of Illinois at Chicago).

^2^Cardioedit software (Brain-Body Center, University of Illinois at Chicago).
